# Smart FL: meta-learning for federated blood marrow smear classification

**DOI:** 10.3389/fonc.2026.1841558

**Published:** 2026-07-09

**Authors:** N. Ilakiyaselvan, Srivastava Sanskar, D. Aarthi, V. Kalyanasundaram

**Affiliations:** School of Computer Science and Engineering, Vellore Institute of Technology, Chennai, Tamil Nadu, India

**Keywords:** bone marrow cell classification, FedAvg, federated learning, meta-learning, prototypical networks

## Abstract

Accurate classification of bone marrow cells is crucial for diagnosing hematological disorders, yet integrating machine learning and deep learning models into clinical practice faces hurdles like data privacy and data scarcity. Existing models often lack the adaptability that is important in the medical field, limiting their practicality. This study introduces an approach using federated learning and meta-learning to tackle these challenges. The objectives for this study were multifaceted. Firstly, it address the adverse effects of data scarcity commonly found in medical datasets due to which models are unable to predict classes which do not have enough examples. Secondly, this study tackles the privacy concerns by adopting a federated learning framework, allowing model training without centralizing sensitive data. Additionally, the goal is to make the federated process personalized to each client for enhancing individual accuracy. Lastly, this study seeks to improve the generalization capabilities of the models, enabling robust performance across diverse patient populations. The proposed approach involves leveraging a ResNet-18 backbone for the meta-learning algorithm, specifically one based on prototypical networks. This framework is then implemented and tested in a federated manner using FedAvg across four clients, each possessing their own data. The main focus of this research is to achieve high accuracy, particularly in unseen classes with limited samples. This approach yields promising results, achieving an accuracy of 96 ± 1% in classes with low sample sizes. Through this approach, hospitals, clinics, and researchers can make sure that their data remains private while being able to benefit from deep learning research.

## 1 Introduction

The classification of bone marrow cells holds immense significance in the realms of hematology and clinical medicine. It is crucial for accurately diagnosing various hematological conditions like leukemia, lymphoma, and myelodysplastic syndrome. Being able to distinguish between normal and abnormal cell populations is vital for clinicians to make well-informed decisions about patient health and the most suitable treatment options. Often, treatments are tailored to each individual patient based on the specific abnormalities identified. Additionally, tracking these cell populations over time provides valuable insights into how diseases progress. Despite the availability of other detection methods, bone-marrow cytology remains essential because it is quicker and more technologically accessible to larger populations compared to newer methods. This method typically involves experts examining cell morphology under a microscope and manually classifying them. One major hurdle is the multiple number of cell classes they could belong to. Manual assessment is time-consuming and limited by human ability, which means the accuracy of classifications depends on the examiner’s expertise. This also makes it difficult to identify rare cell types.

In recent years, the medical field has witnessed a surge in efforts to automate various processes, courtesy of advancements in AI and deep learning. Image classification, in particular, has seen remarkable progress, thanks to the emergence of convolutional neural networks (CNNs). While CNNs have been successfully applied to numerous medical images, much of the research has focused on prevalent diseases with ample data. This preference stems from the fact that CNNs demand vast quantities of high-quality images and annotations to yield effective results. Consequently, acquiring appropriate data for less common diseases or conditions, where research data may be scarce or privacy concerns impede data sharing, proves challenging. Moreover, labelling extensive datasets can incur substantial expenses, exacerbating the issue.

In recent studies, various approaches utilizing deep learning CNN models have been explored for the classification of bone marrow cell images. The ensemble classifier described in one study (12) comprised two ResNet models, achieving validation accuracies of 82% and 88%, respectively, for single-cell image classification in bone marrow samples. However, as noted in the findings reported in another study (13), classification rates varied among cell classes, with some classes achieving high average precision (AP) values of 97% and 92%, while others exhibited lower AP values of 60% and 54%. The observed discrepancies were attributed to potential class imbalances or cytological heterogeneity within rare cell types. A study reported in another source (14) utilized an AI-based platform for bone marrow smear classification, achieving an accuracy of approximately 87.50%. However, the model’s sensitivity and specificity were noted to be 69.40% and 97.20%, respectively, indicating challenges in accurately identifying false negatives due to nature of the data.

Furthermore, researchers in another investigation (15) employed an EfficientNetV2S backbone CNN model pretrained with weights, achieving robust performance with an area under the receiver operating characteristic curve (AUROC) of 95% for 11 cell types. Nevertheless, issues persisted in accurately classifying other cell types, particularly abnormal/artifact cells and along the myeloid lineage, such as blasts, promyelocytes, and myelocytes. Similarly, another study (11) utilized a ResNext-50 model with data augmentation, encountered challenges related to class imbalances, resulting in varying precision values ranging from 17% to 95% depending on the cell type. Collectively, these findings reveal a common trend among deep learning and CNN models, where satisfactory accuracy is achieved for most classification tasks, but challenges persist in accurately classifying classes with limited examples. Thus, while achieving high accuracy for classes with abundant examples is notable, there is a critical need to improve accuracy for classes with limited representation.

This predicament gives rise to two primary challenges: privacy preservation and data scarcity for training. Federated Learning offers a potential solution by decentralizing data across multiple sources, enabling collaborative model training without data exchange. Through this approach, hospitals and clinics can safeguard the privacy of their data while harnessing the benefits of deep learning research. For instance, consider a scenario where five clinics each possess datasets on bone marrow cell classes. Individually, these datasets might be insufficient for model training. However, leveraging federated learning principles allows each clinic to train a central model using their data, sharing only model updates for averaging and subsequent refinement. However, this method introduces another challenge. Since it involves averaging the weights, we would obtain the best average model but not the best model for each individual client. This highlights the need to address the issue of personalization and generalization. In cases like COVID-19, where multiple strains exist, or in the context of bone marrow cytology where new cell classes may emerge, ensuring model adaptability becomes paramount. Meta-learning, or learning to learn, offers a promising solution. By leveraging insights gleaned from previous data to adapt to novel, unseen data, meta-learning excels in personalization and adaptability. Integrating these benefits into our approach allows us to address all concerns effectively. Thus, the main objectives of the study are:

Multiple researchers (1) have noted that conventional federated averaging (FedAvg) methods may lead to decreased accuracy, with reported values as low as 75%. However, by incorporating personalization techniques, such as the use of the Model-Agnostic Meta-Learning (MAML) algorithm, they were able to significantly improve accuracy to 90%. Notably, this highlights the importance of balancing global accuracy optimization with personalized model adaptation. Similarly, another set of researchers (2) proposed a variant called “Per-FedAvg,” leveraging meta-learning frameworks to enhance the central model’s ability to personalize to local user data. Their findings also demonstrated an increase in accuracy following the implementation of this approach. Meanwhile, certain researchers (3) and their colleagues have explored the trade-offs between convergence and accuracy in local update models like meta-learning and FedAvg, shedding light on the complexities inherent in optimizing these techniques. Moreover, other studies (16) have verified that the integration of meta-learning and federated learning techniques does not compromise privacy but in fact strengthens it. Additionally, researchers (5) have observed that incorporating certain meta-frameworks can increase the speed of model convergence while simultaneously enhancing accuracy. Collectively, these studies helped us realize the importance of addressing personalization challenges in federated learning approaches, with various techniques showing promise in improving model accuracy and adaptation to local data characteristics. However, ongoing research is needed to further explore and optimize these methods for real-world applications. The main objective of this study is to identify an optimal starting point from which the model can swiftly adapt to individual data, thereby facilitating efficient classification and adaptation.

This research aims to explore the potential of federated learning and meta-learning methodologies in the precise classification of bone marrow cells. and the process are as follows:

- Comprehensive review of relevant literature in section 2.
- The technical understanding of federated learning and meta-learning, respectively, explaining their significance and applicability to our specific research objectives in sections 3.1 and 3.2.
- In the subsequent section, we detail our methodologies and materials, outlining various aspects such as our dataset selection, augmentation techniques employed, the implementation of prototypical networks, utilization of the FedAvg algorithm, selection of the CNN backbone architecture, and the setup of our experiments in Section 4.
- The core of our study unfolds in the results and discussion section (Section 6), where we present our empirical findings and engage in a thorough analysis of the outcomes. Here, we scrutinize the implications of our results, discussing their significance in the context of our research objectives and shedding light on any notable observations or trends uncovered during our experiments.
- *Mitigate Class Imbalances*: Use meta learning to mitigate the impact of imbalanced data in blood marrow smear classification.
- *Address Privacy concerns*: Use federated learnings decentralized system to secure the data.
- *Achieve Personalization*: Implement Meta Learning to achieve personalization for local devices within the federated learning setup.
- *Achieve Generalization*: Implementation of the model should be able to generalize on new unseen classes, this is supported by using data augmentation.
- Finally, we conclude our study’s key findings in Section 7,reiterating its contributions to the field and outlining potential avenues for future research.

## 2 Related works

Numerous research papers have delved into the challenges outlined earlier, particularly in the realm of medical data analysis using deep learning frameworks but based on the author’s perspective the following research papers have been chosen to be used for this study. Junghwan Lee et al. had explored the role deep learning plays in the advancement of rare disease research (1). For this the researchers have reviewed over 332 articles pertaining to this topic. Apart from understanding that majority of the research regarding this utilize CNNs, the researchers have also concluded that lack of data is cited to be the predominant challenge in 43.40% of articles. Researchers like Tongnian Wang et al. had noticed that deep learning has fueled the growth of multiple health applications. The research that is being done in this field faces many issues that hinder its usage in the real world (2). These are privacy concerns and lack of data, which hinder the maximum number of researchers. They have proposed federated learning as one of the solutions to these problems. This allows for collaborative training without the issue of sharing data. They have researched 26 papers which highlight the applications that such training could have on healthcare, focusing primarily on the remote monitoring and diagnosis. The review also identifies the challenges federated learning has to face like expensive communication, statistical heterogeneity and system heterogeneity along with solutions for each. The paper does lack the explanation and solution for the problem of personalization. Similarly, Anichur Rehman et al. had also explored the potential that federated learning has but extended the field of its integration to deep learning, Artificial Intelligence, Explainable AI and many more. The researchers have examined the technologies and how they could be integrated with each other especially in the domain of healthcare, addressing challenges and potential solutions (3). Challenges such as security, privacy, stability, and reliability have been focused on thus guiding the readers towards the solutions provided by federated learning.

Bingyang Chen et al. had realized the issue that federated learning faces with respect to personalization. Their research is geared towards rare disease prediction where federated learning offers a potential solution to the privacy and data scarcity (4). The approach utilized by them involves the usage of Model Agnostic Meta Learning (MAML), attention-based mechanisms and dynamic weight fusion for enhanced disease prediction. This method dynamically adjusts the attention to the more challenging tasks. Alireza Fallah et al. also integrated the principles of model agnostic meta learning into the working of federated learning to make a more personalized variant of federated learning. This research is more geared towards the theoretical guarantees (5). The researchers state that the goal is to find an initial model that the new users can adapt easily to. This is done by using their Per-FedAvg variant which reaches an adaptable model state which allows the model to personalize to new users after a few local gradient steps. They have also compared their method with conventional models to show the improvements. Zachary Charles et al. have delved into the group of algorithms known as local update methods that offer a potential solution to the problem faced by federated learning. This paper offers a generalized perspective encompassing various federated learning approaches (6). Quadratic models are used, establishing the equivalence between local update methods and first-order optimization on the loss. The paper also considers the trade-off between accuracy and convergence and provides a method to understand these critical points.

Fei Chen et al. addressed the statistical and systematic challenges faced while employing federated learning, particularly across distributed networks of mobile devices. Their proposed solution Fed-meta employs a meta learning framework which involves the parameterized algorithm being shared among clients (7). This was then tested on LEAF dataset and a real-world production dataset showing its effectiveness. While using meta learning frameworks enhances the general performance of the models and helps in personalization, there is still the matter of privacy concerns. Omid Aramoon et al. explores this by analyzing the effects that such integration may have on the security provided by the traditional federated learning (8). For this he has focused on the possibility of defending against backdoor attacks when the secure aggregation is in place. His approach, the Meta-FL is a version of integrated meta learning and federated learning that is compatible with secure aggregation. The research systematically evaluates the model on two datasets and compares its utility and robustness against adversarial attacks as compared with normal FL. He concludes that such integration is not detrimental but enhances the defense. Many of the issues like security and personalization have been addressed and Yihan Jiang et al. had also contributed towards this by aiming to deal with the heterogeneity in such situations including personalization for federated learning. To increase the rate at which the model is able to optimize and adapt they have also used model agnostic meta learning (9). The researchers have also noticed that the traditional FedAvg algorithm can be interpreted as a meta learning algorithm on its own. Thus, even regular fine-tuning can yield better results and accuracy. Therefore, it is imperative to not only focus on the global accuracy but also the personalized results. There is also the issue due to unbalanced data distributions between clients which can be solved using the meta learning techniques. For each task in the process the algorithms use a variant of gradient descent locally and send the update to the coordinator. The researchers have presented the federated training process as meta training and federated personalization as meta testing. This is a modification of the FedAvg with 2 stages if training and then fine-tuning. The researchers have also explored the usage of Reptile algorithm for meta learning instead of the popular MAML.

Bokun Wang et al. had recognized the popularity of model agnostic meta learning while realizing that the stochastic optimization for MAML remains under developed. The traditional algorithms face challenges in the convergence and stability aspects. This poses an issue when integrated with continual or cross-device federated learning. This research thus proposes memory based stochastic algorithms for MAML and addresses the challenges mentioned (10). These novel algorithms are MOMLv1 and MOMLv2 with LocalMOML for federated settings. These algorithms are improvement on both optimization theory and empirical results. They are evaluated on sine-wave regression and similar one-shot classification tasks. Renkun Ni et al. had meanwhile focused on the issue of imbalanced datasets instead. Even though meta learning and its variants perform impressively even with imbalanced datasets there are always ways to improve the learning capabilities of the models. One way this can be done is using data augmentation (11). Data augmentation is artificially increasing the data available for testing thus making the model easy to generalize which is necessary for meta learning. In meta learning, where we divide the available dataset into tasks consisting of support and query sets, data augmentation becomes more nuanced. The study systematically explores the entire meta learning pipeline, exploring how data augmentation affects the process when used at different stages. We are introduced to meta specific data augmentation which focuses on meta learning’s sensitivity to query data which indicates that it is more important to augment query than the support set. This is the proposed Meta-MaxUp which combines various met-specific data augmentations for maximum improvements.

Christian Matek et al. had worked on the same data chosen by the author. Their study addresses the manual classification of bone marrow cell cytomorphology by applying convolutional neural networks to a substantial dataset of 171,374 cytological images from bone marrow smears (12). The model they have used is a pre-trained ResNeXt-50 and exhibits accurate prediction performance on the relevant cell species. Limitations include reduced performance for classes with limited training data, requiring more data for improved recognition. Explainability methods like Smooth Grad and Grad-CAM confirm the network’s focus on relevant regions and characteristic features of specific cell classes. UMAP embedding analysis verifies the network’s ability to stably separate morphological classes and tolerate label noise. Future work may further address label noise relevance through semi- or unsupervised methods.

Jacqueline Kockwelp and others, had developed a new deep learning pipeline to quickly and cheaply predict genetic alterations from single cell images taken from standard stained bone marrow smears taken at diagnosis (13). Using CNNs, the authors compiled a large dataset of greater than 2 million single cell images from diagnostic samples from 408 patients. We have a one stop solution where the CFM can scan the images of bone marrow to extract single cell images, classify the images to different classes and then predict for those genetic indicators that are important.

Rohollah Moosavi Tayebi et al. developed an end-to-end deep learning based approach for the automated assessment of bone marrow cytology. The approach begins with a digital whole slide image (WSI) of a bone marrow aspirate and quickly assesses the WSI to determine appropriate regions for cytology assessment, then it detects and classifies all of the bone marrow cells in each of the regions (14). This body of cytomorphological information is packaged as the Histogram of Cell Types or HCT and represents a cytological patient fingerprint. To accomplish these aims the researchers in this study implemented a fine-tuned DenseNet model to quickly assess appropriate regions of interest from the WSI. After those appropriate locations were assessed, a YOLO model was trained from scratch to detect and classify all cellular and non-cellular objects. The cytological information was then summarized for each patient as the HCT, which represented the class probability distribution across each of the types of bone marrow cells. Xinyan Fu et al. developed a new artificial-intelligence based approach for automating the classification of bone marrow cells, and to encourage exploration of practical application for clinical uses (15). Xinqiao Hospital provided the bone marrow smears. The Morphogo automated analysis platform created whole digital images of the bone marrow smears. Subsequently analysed by the AI platform. This Morphogo system is comprised of CNNs made up of 27 layers and was trained on greater than 3,000 bone marrow smears. It is currently able to classify more than 45 bone marrow cell types.

Joshua E. Lewis et al. created a fully automated machine learning pipeline for obtaining accurate 11-component differential cell components (DCCs) from whole slide smears (16). This pipeline processes images to identify ideal regions, automatically detects each, and then classifies each nucleated cell in the marrow into the 11 DCC component using convolutional neural network models deriving from a garnered dataset comprised of manual annotations. The pipeline DCCs show consistent agreement with manual counting identified across a wide variety of pathology bone marrow smears.

After going through quite a few research papers we can start seeing a pattern, most of the models used work very well on most of the classes except those few classes where either there is a lack of enough labelled examples or the classes are similar to one another. Federated learning can address the lack of data and privacy concerns but needs to be personalized to get a model that can accurately perform predictions. **Table 1** summarizes the major existing studies related to federated learning, meta-learning, and bone marrow smear classification techniques discussed in the literature.

**Table 1:** Summary of related work.

Author	Method	Result/findings
Junghwan Lee et al., 2022 (1)	Scoping review exploring the role of deep learning in advancing rare disease research.	Insufficient data is the predominant challenge cited in 43.40% of articles.
Tongnian Wang et al., 2023 (2)	Review of multiple papers highlighting Federated learning applications.	Identifies challenges like and proves to be effective in mHealth applications.
Anichur Rahman et al., 2023 (3)	Federated learning with AI, XAI.	Integration techniques and challenges for different approaches.
Bingyang Chen et al., 2021 (4)	Federated Learning with attention-based mechanisms and MAML.	13.28% increase in average prediction accuracy compared to local hospital models.
Alireza Fallah et al., 2020 (5)	Per-FedAvg using MAML framework.	Increase of 5-10% versus regular FedAvg.
Zachary Charles et al., 2021 (6)	FedAvg using MAML.	Tradeoffs between convergence and accuracy in local update methods.
Fei Chen et al., 2019 (7)	Fed-Meta using MAML framework.	Reduction in needed communication cost by 2.82-4.33 times, and an increase in the accuracy by 3.23% - 14.84% compared to FedAvg.
Omid Aramoon et al., 2021 (8)	Meta federated learning using MAML.	Security against backdoor attacks enhanced.
Yihan Jiang et al., 2020 (9)	FedAvg using Reptile meta learning and MAML.	90% accuracy.
Bokun Wang et al., 2023 (10)	Federated learning with novel LocalMOML model.	94% accuracy on one shot with sine wave regression.
Renkun Ni et al., 2021 (11)	Meta-learning specific data augmentation- Meta-MaxUp.	Findings indicate meta-learning’s sensitivity to the amount and quality of query data.
Christian Matek et al., 2021 (12)	ResNeXt model.	20-96% (class wise) precision and recall.
Jacqueline Kockwelp et al., 2024 (13)	Novel Deep learning pipeline using 2 ResNet18 as ensemble.	Accuracies of 0.82 and 0.88 for single cell classification.
Rohollah Moosavi Tayebi et al., 2022 (14)	DenseNet121 classifier.	The Cell classification ranges from 0.64-0.93. (0.75 mean average precision, 0.78 average F1-score, Log-average miss rate of 0.31)
Xinyan Fu et al., 2020 (15)	Software utilizing an AI-based platform.	The Classification accuracy was above 85.70%. And the Averages of sensitivity and specificity of the system were found to be 69.40 and 97.20%, respectively.
Joshua E. Lewis et al., 2023 (16)	Pipeline using multiple CNN networks.	Accuracies 95% and above for normal classes with poorer performance on classes with low samples.
Jake Snell et al., 2017 (17)	Prototypical network trained on Omniglot and ImageNet.	Accuracies reach 98%.

## 3 Technical background

As we have discussed before, traditional image classification tasks rely on centralized data repositories and expert annotations. Due to this, these tasks face challenge concerning data privacy, scalability, and model generalization. In recent years, federated learning has risen as a promising method for collaborative model training across decentralized data sources, addressing these concerns by enabling model training directly on distributed data while preserving data privacy. Meanwhile, meta learning techniques offer the ability to leverage prior knowledge from multiple tasks or domains to facilitate learning and adaption to new tasks, potentially enhancing the robustness and efficiency of classification models. In this context, the integration of federated learning and meta learning presents a novel approach to image classification tasks, harnessing the collaborative power of federated learning while leveraging meta learning to improve model performance across heterogeneous datasets and tasks. In this section an overview of federated learning and meta learning concepts, are highlighted and in **Table 2** Intermediate Accuracy of Different Meta Learning Methods are show.

**Table 2:** Intermediate accuracy of different meta learning methods.

Method	Intermediate accuracy
MAML- Model Agnostic Meta Learning	48%
Reptile	52%
Prototypical Network	70%
Episodic Prototypical Network	73.5%

MAML (Model-Agnostic Meta-Learning) aims to find model parameters that can be easily adapted to new tasks with a few gradient steps. It learns a good initialization that allows for rapid adaptation to new tasks. MAML represents a model by a parameterized function *f_θ_*, where *θ* denotes the parameters. When adapting to a new task *τ*, the model’s parameters *θ* are updated to *θ*′ using gradient descent on task *τ*. MAML optimizes the model parameters to ensure that a few gradient steps on a new task result in highly effective performance, using stochastic gradient descent (SGD) for optimization.
Reptile is a straightforward meta-learning algorithm that learns a parameter initialization for fast adaptation to new tasks. Unlike MAML, Reptile does not require differentiating through the optimization process. Thus, it is more appropriate for many update steps. Reptile repeatedly samples a task, trains on the task, and shifts the initialization towards the weights it learned in that task. By learning an initialization for the parameters in a neural network model, Reptile can quickly learn from a few examples of a test task. In the last step, instead of simply updating the parameters *θ* in the direction of *W* − *θ*, Reptile treats (*θ* − *W*) as a gradient and incorporates it into an adaptive algorithm like Adam, enhancing the efficiency of the learning process.

### 3.1 Meta learning

Meta learning, also known as learning to learn, is a fascinating concept that aims to mimic the way that humans acquire new skills and knowledge. At its core, meta learning is about teaching models to become better learners themselves. Imagine if you could learn how to learn more efficiently, making it easier to pick up new skills or adapt to new situations – that is what meta learning aims to achieve. Any machine learning or deep learning model requires a large number of samples during training while humans can learn new concepts and skills much faster and more efficiently. A human can learn to distinguish between a cat and a dog with just a few examples, a person who has learnt one instrument can learn the second one easier. In technical terms, meta learning refers to a set of techniques aimed at enabling models to generalize across tasks or datasets by leveraging prior knowledge acquired from related tasks. This adaption process is similar to the training phase at a smaller scale. A strong meta-learning model ideally undergoes training across a diverse array of learning tasks, each accompanied by its own dataset. Instead of iterating over the entire dataset in one epoch, meta learning aims to train in the same way as testing.

To do this meta learning aims to divide the Datasets into smaller tasks which are then treated as training and testing sets. Each task is comprised of a support set and a query set. The support set acts like the training data while the query set acts like the testing data. The term often associated with such learning is called K-shot N-way classification, where K represents the number of labelled examples for each of N classes in the support set. Suppose you want to train a model to recognize different species of flowers. In a 3-way 2-shot tasks, the support set would contain two labelled examples of 3 different flowers species, while the query set would contain several unseen images of those species. Our goal is to reduce the error on the samples of unknown classes given a small support set.

**Figure 1:**
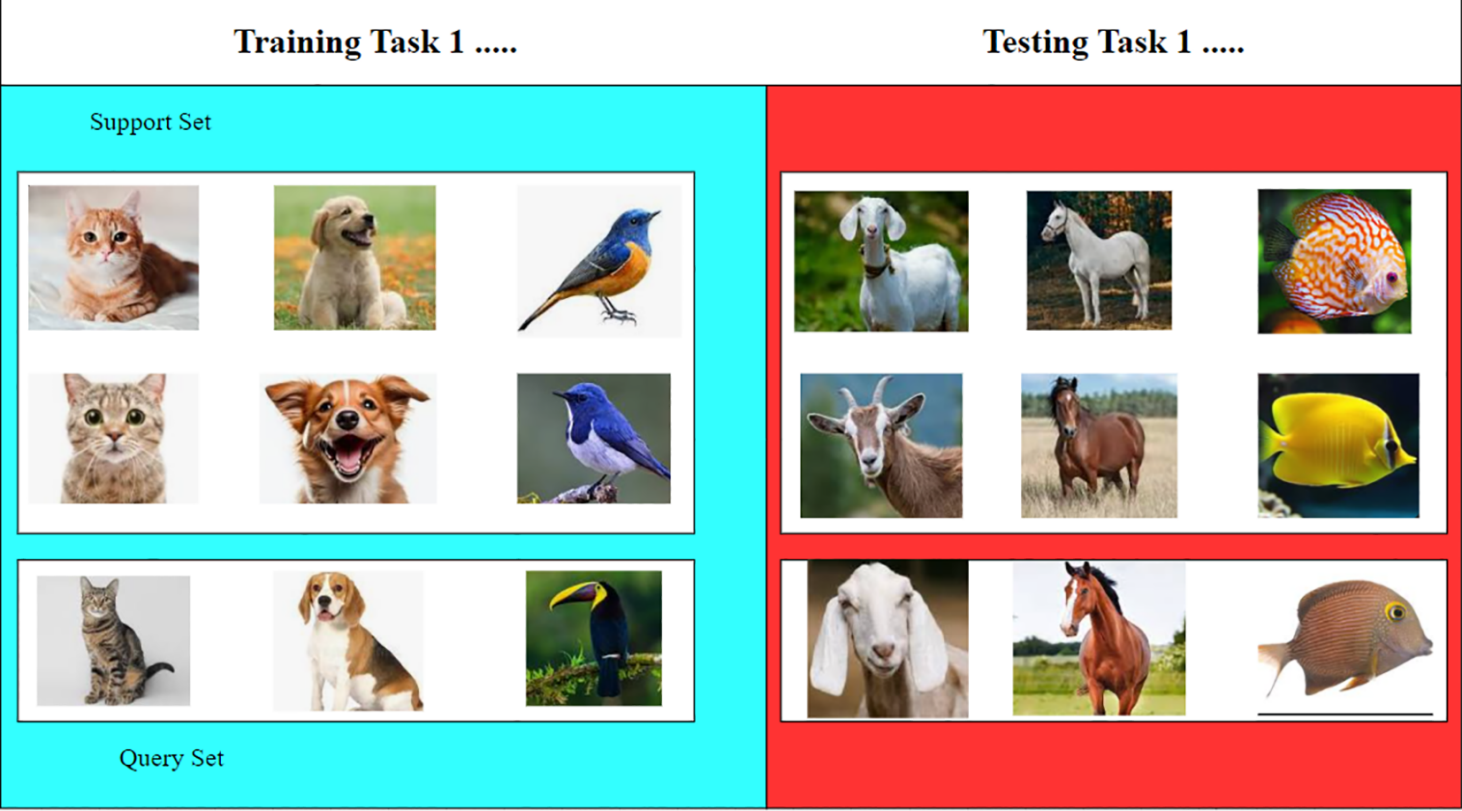
Meta Learning 3-way 2-shot.

**Figure 2:**
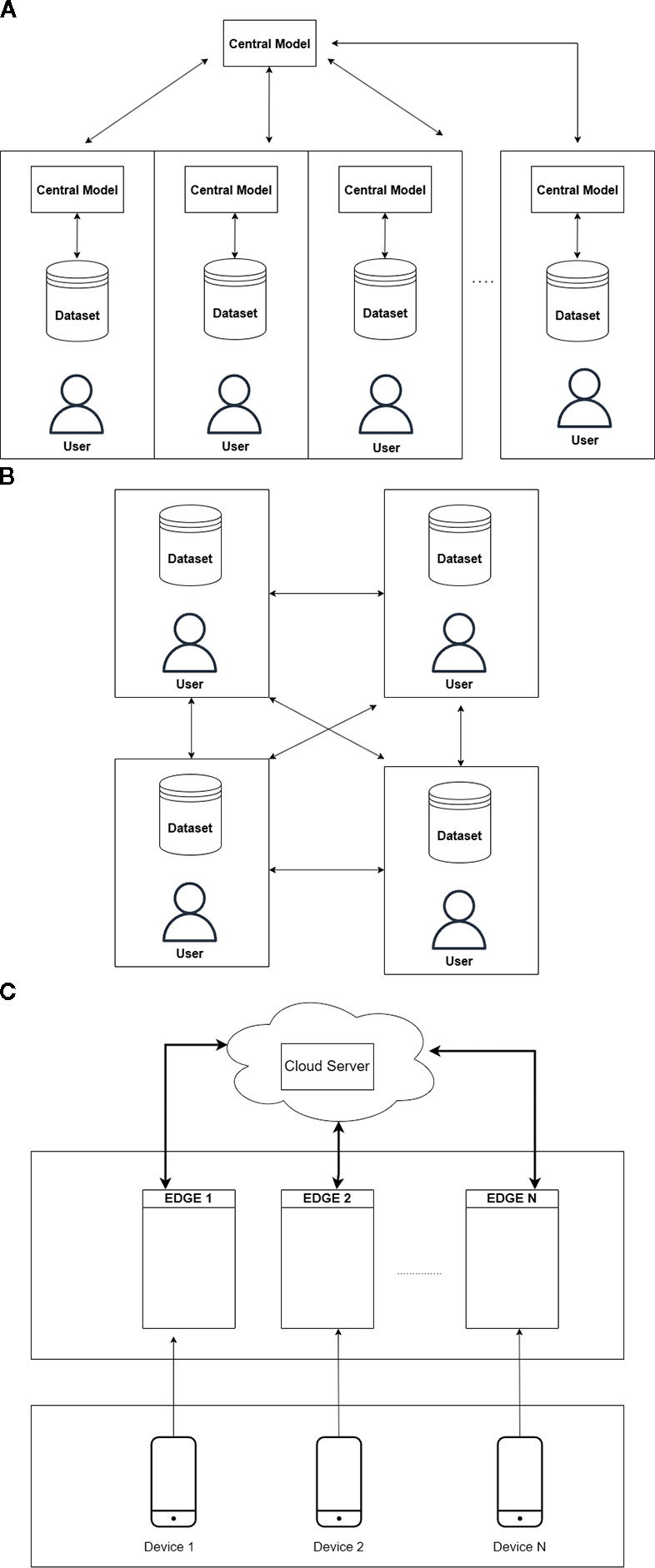
Types of federated learning architectures: **(a)** Centralized, **(b)** Decentralized, and **(c)** Edge based Federated Learning.

As you can notice from the **Figure 1**, the training and testing task both have 3 classes and 2 images in the support set. This is another 3-way 2-shot classification task. The above model would train on the examples provided by the support set and then test what it learned on the images present in the query set. Meanwhile the training and testing tasks have disjointed sets of classes i.e. there is no overlap within each task. This is to make our model adaptable and easier to generalize for unseen classes. This means unlike in traditional machine and deep learning where we divide the dataset based on the number of data samples e.g. 80–20 spilt, in this case there is a division in the dataset based on the classes. **Figures 2a–c** represents the different types of federated architectures. Different classes are included in both the training and testing tasks. Doing this we aim to make the model be able to handle any new unseen classes. After we get a good adaptable model, we can then fine-tune it on the required data containing the unseen classes to personalize it to the user’s preference. There are many approaches to meta learning, the most popular ones are known as metric based, optimization based and model based. In this research we have utilized a technique belonging to metric-based learning.

#### 3.1.1 Metric learning

Metric based meta-learning is conceptually similar to the traditional algorithms like k-means clustering. Much like k-means, where data points are grouped into clusters based on their proximity to centroids, metric-based learning aims to classify data points by computing their similarity to the support set samples. Let us consider a scenario where we have a set of customer transactions, where each transaction is represented by various features such as purchase amount, frequency of purchases and customer information. In k-means clustering, the algorithm iteratively assigns each transaction to one of k clusters based on the similarity of their features. Transactions with similar purchase amounts and frequencies may be grouped together in one cluster, while transactions with different characteristics would belong to another. Drawing parallels with metric-based meta-learning, in this context instead of customer transactions, we have a support set consisting of samples of different customer segments. When a new transaction comes in, the model evaluates its similarity to the transactions in the support set using a kernel function. By measuring this similarity the model can classify the transaction into the appropriate segment. In general the predicted probability over a set of labels is related to the weighted sum of the labels in the support set. This weight form the labels is calculated using a kernel function which measures the similarity between the data samples. The success of the model depends on its ability to learn a good kernel.

### 3.2 Federated learning

Federated learning is a transformative approach to machine learning, particularly in scenarios where data privacy is of the utmost importance. By decentralizing the traditional machine learning process, it not only addresses the privacy concerns but is also able to leverage the collective intelligence of distributed devices to improve the model accuracy. Unlike conventional methods that centralize data on a server, i.e. the model and the data usually are on the same device, federated learning allows the collaborative training between multiple clients or edge devices. This means that the model and local data are no longer present in the same device. The edge devices are enabled to train the model on their respective data and only share the model updates with the central server. This allows the model to learn from diverse and potentially biased data sources making the model adaptable and generalizable.

This type of training ensures that the privacy of the individual data points is maintained. This is particularly advantageous in situations where data cannot be easily centralized due to regulatory constraints or privacy concerns. For instance, in the domain of healthcare, federated may allow the hospitals or clinics to collaborate and utilize their own data to train medical AI models without having to share the private patient data, thus ensuring compliance with data protection regulations like Health Insurance Portability and Accountability Act (HIPAA). Moreover, federated learning is an iterative process which enables continuous training and adaption to changing data distributions without the need of centralized retraining. This is a crucial advantage in dynamic environments where data distributions may shift over time, such as online retail or social media platforms. By allowing the models to be updated locally on devices and then aggregated centrally, federated learning facilitates real-time adaption to evolving user preferences and behaviors.

Federated learning is being used across various domains with great results.

- Healthcare: Google’s Deep Mind Health division is exploring federated learning for healthcare applications.
- Smart Devices and IoT: Apple and Google are known to use federated learning on their smartphone environments and other smart devices. Apple uses it in its iOS ecosystem for improving tasks like predictive text and suggestions on its keyboards.
- Finance: American Express utilizes federated learning to enhance fraud detection capabilities while protecting customer privacy.
- Telecommunications: Nokia is researching and implementing federated learning techniques to optimize network performance in telecom infrastructure.
- Autonomous Vehicles: Tesla also utilizes federated learning to continuously improve its autopilot system.

Most federated learning approaches involve the same iterative process of local training, model update sharing and aggregation. Models differ based on how the model is shared and aggregated.

Some of the types are.

- Centralized Federated learning: In centralized federated learning, there is a central server which is responsible for aggregating model updates from the clients. The training process involves devices locally training their models on their data and then sending the updates to the central server. The central server aggregates the updates to update the global model, which is then redistributed to the devices for further refinement.
- Decentralized Federated Learning: This type of federated learning is also known as peer-to-peer federated learning, it eliminates the need for a central server as the devices directly communicate with each other to exchange model updates. This necessitates the presence of efficient communication architecture and protocols among the participants.
- Edge Federated Learning: This type leverages multiple edge devices such as smartphones, IoT devices etc. to perform the model aggregation.

For the sake of deeper understanding, we will explain centralized federated learning. Let us consider a real-life example in the context of improving voice recognition in smart speakers. The goal is to improve the accuracy of the voice recognition system while maintaining user privacy. In the traditional approach, the company would have to collect the voice data from users, send it to a central server for training and processing. This obviously makes people hesitant as their voices would be shared with a third party. To address these concerns, the company adopts federated learning. Federated learning can be realized using three architectural paradigms, namely, centralized, decentralized, and edge federated learning architectures. Centralized federated learning requires the presence of a central server that aggregates models across all clients. Decentralized federated learning does not require a central server to coordinate communications between clients. Edge federated learning leverages edge devices for aggregating and computing models in a distributed manner.

The process goes as follows –

•Initialization: The company initializes a global model which can be used for voice recognition and a copy of this model is sent to all the clients/clients possess a copy of the global model.
•Local Client Training: Each user’s smart speaker locally trains a personalized voice recognition model using the voice it collects from the user. For examples in the figure considers User 0, the training will occur on the smart speaker owned by the user on that device. This is done to make sure that the user’s voice does not leave their device.
•Model Aggregation: Periodically, the smart speakers send only the updates made to their local models and not the raw data to the local server. These updates are then aggregated to improve the model.
•Iteration: The updated global model is then redistributed to all the smart speakers, where the process repeats. This continues till the model reaches convergence or up to a pre specified condition.

This approach does require high local memory capability and computing power with sufficient bandwidth connections. This is because federated learning can involve multiple devices in one network. Lack of resources or bandwidth may cause the transfer of the updates to become slow. There are multiple techniques to reduce the size of the messages so that transfer speed is maintained. The multiple clients in federated learning can also belong to systems with different specifications. This introduces issues of system heterogeneity where different clients have differences in storage, communication, and computational resources. While techniques that tackle the above issues are prevalent, we will focus on another issue in our research. Statistical heterogeneity occurs in federated learning when the clients themselves have a variety of distributions among the local datasets. Since the main concept of many federated learning algorithms involves taking the average of the model parameters, this makes the final model the best average model overall but not the ideal model for each client. This is the issue of personalization which can be addressed using local update methods which adapt the global model to the local client.

### 3.3 CNN backbone

In our experimental setup we have utilized a backbone for our meta learning algorithm. ResNet18 or Residual Network 18 is a popular convolutional neural network architecture. Residual Networks were introduced by Kaiming He et al. in their paper “Deep Residual Learning for Image Recognition” (2015). It included an innovative approach in its residual blocks. These blocks allowed for the training of deep networks by mitigating the vanishing gradient problem. ResNet18 as the name suggest belongs to this family of models and has 18 layers, including convolutional layers, batch normalization, and fully connected layers. The aim is to learn residual functions which represent the difference between input and output of a layer. These residuals are then added back to the input, effectively skipping layers during training. This allows the network to learn both the identity mappings and the more complex transformations as well. We leveraged the pretrained ResNet18 backbone from the torchvision library provided by Pytorch. This backbone has already learned rich feature representations from the massive ImageNet dataset. By using this we benefit from its ability to extract high-level features. This will also serve as powerful initialization point so that we can achieve the best adaptable model relatively quickly in the global training phase. We have then integrated this with our model. Within our network we compute prototypes or kernel function as mentioned in the metric learning section for every class based on the feature representation extracted by the ResNet18. During inference we compare the input samples to these prototypes to make decisions. After passing through the fully connected layers of the ResNet18 we applied a flattening operation which reshapes the output tensor into a 1-D vector, preparing it for subsequent processing.

## 4 Methods and materials

### 4.1 Dataset description

**Figure 3:**
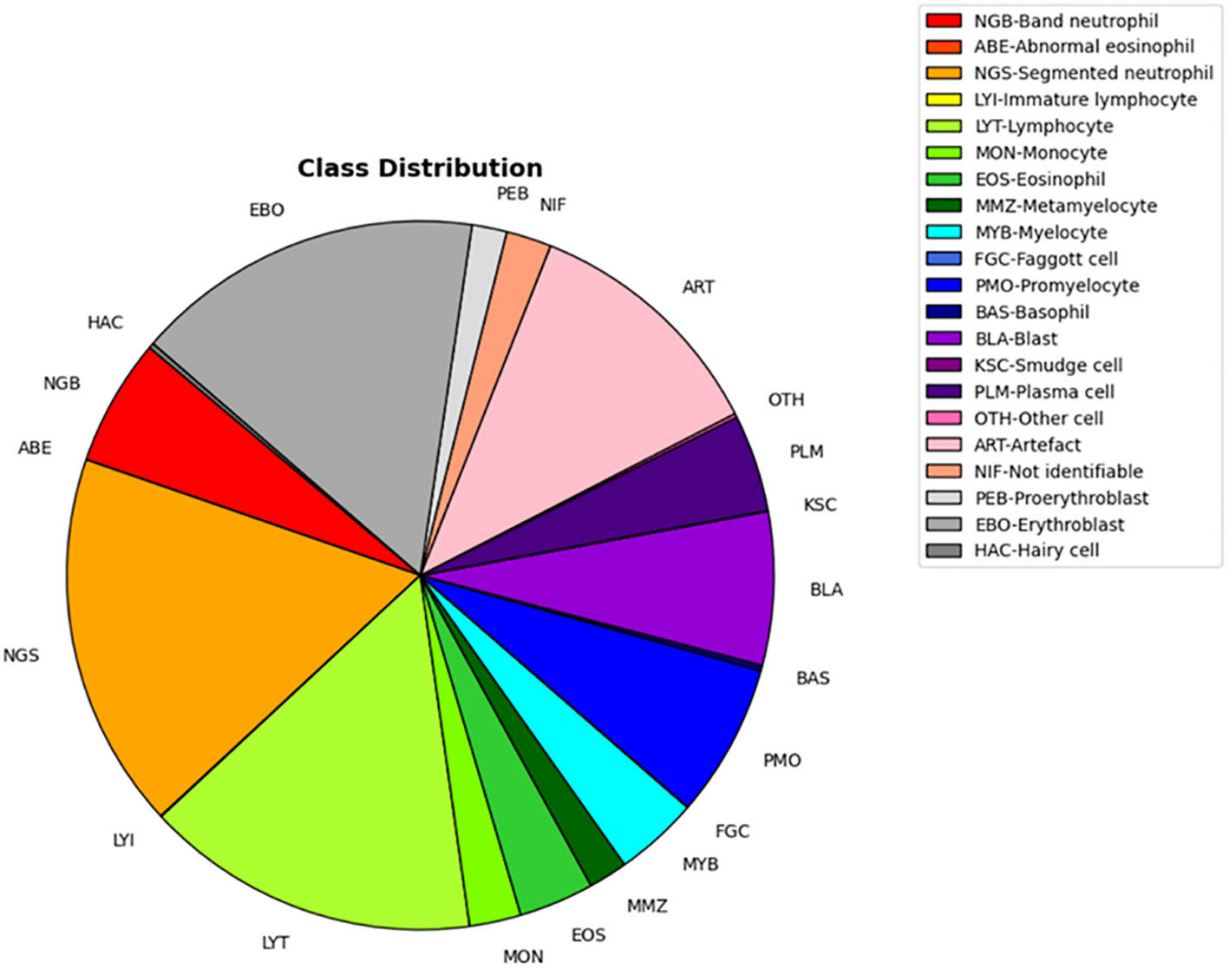
Data distribution of bone marrow cell.

The dataset used in this study originates from “An Expert-Annotated Dataset of Bone Marrow Cytology in Hematologic Malignancies” (18), comprising data collected from 945 patients in JPG format. As bone marrow morphology serves as a crucial diagnostic tool for a wide spectrum of hematologic diseases, this dataset is made available for research purposes, provided proper referencing is adhered to. With 21 distinct classes, the dataset exhibits imbalances as can be seen from **Figure 3**, necessitating careful partitioning for training, validation, and testing phases. This partitioning strategy involves allocating 11 classes for training, 6 for validation, and 3 for testing, with unique classes designated for each phase. Notably, the testing subset is tailored to mirror real-world scenarios by encompassing unseen classes with limited samples, ensuring the model’s ability to generalize effectively. **Table 3** presents the dataset distribution utilized in this study.

**Table 3:** Dataset class distribution.

Dataset component	Number of classes
Training Classes	11
Validation Classes	6
Testing Classes	3
Total Classes Utilized	20

**Figure 4:**
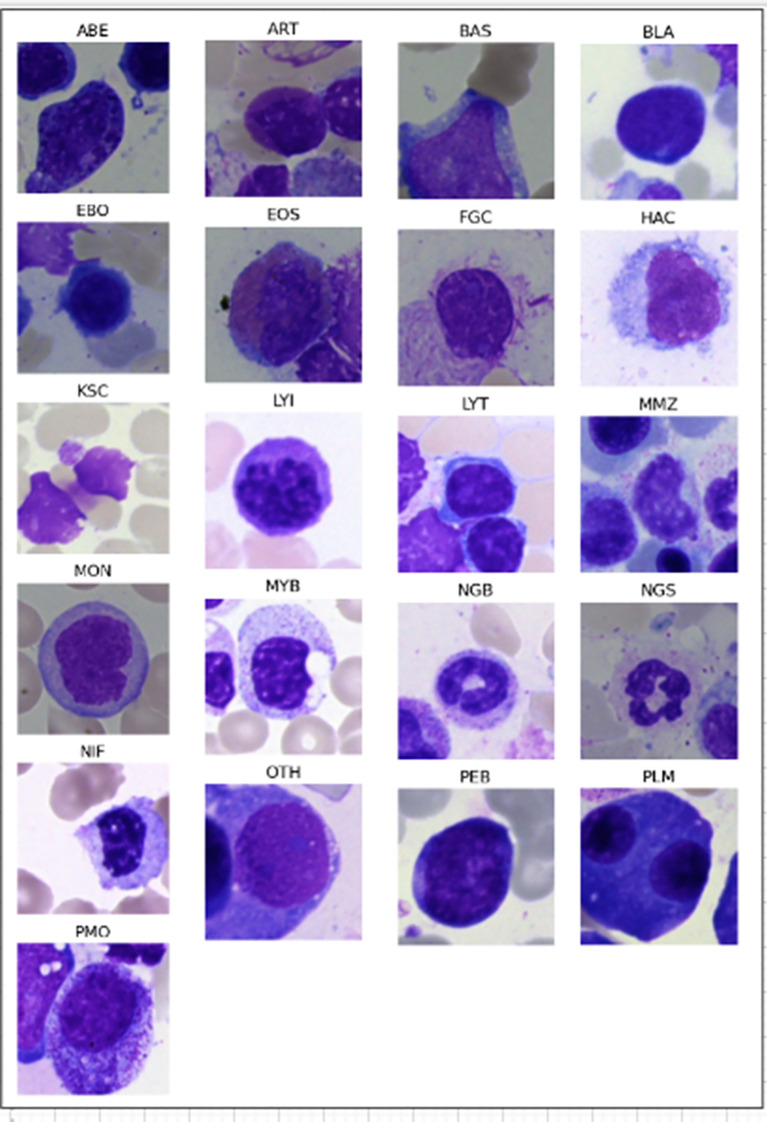
Class representation of bone marrow dataset.

Due to the imbalanced nature of the dataset, separate classes were allocated for training, validation, and testing to evaluate the generalization capability of the proposed framework on unseen classes. All the classes were utilized from the original dataset except one was not included during experimentation due to the task-based episodic partitioning strategy employed during meta-learning and federated evaluation. **Figure 4** illustrates representative samples from the different bone marrow cell classes utilized in the dataset.

#### 4.1.1 Dataset statistics and preprocessing

The dataset used in the current research comes from “An Expert-Annotated Dataset of Bone Marrow Cytology in Hematologic Malignancies” (18), where bone marrow smear images from 945 patients in JPG format were included. The current dataset has 21 classes of bone marrow cells and shows class imbalance among those classes. The size of the dataset is Approximately 6.8 GB.

In order to measure the generalization capability of the proposed framework, the current dataset was divided into three mutually exclusive groups, which include the training class, the validation class, and the testing class. In total, 11, 6, and 3 classes are selected for training, validation, and testing respectively, where the testing data were used for evaluating the adaptability of the proposed meta-learning framework by introducing some unseen classes.

Some data processing steps have been performed, which include applying data augmentation by randomly flipping horizontally, flipping vertically, rotating, and performing affine transformation to all the images in order to enhance the generalization ability of the proposed framework.

### 4.2 Data augmentation

Data augmentation is a crucial technique in the realm of machine learning and deep learning, especially in the domain of computer vision. At its core traditional data augmentation refers to the process of artificially expanding a dataset by applying various transformation to the existing images available. This is done to address the challenge of limited data and enhance the generalization capability of the models. Since one of our core aims is to make our model as generalizable as possible, we have also implemented some basic data augmentation techniques that work for medical images. There exist many types of data augmentation techniques, each designed to introduce variability and diversity in their own way. While trying the different types on our dataset we noticed that the techniques that involved the alteration of spatial orientation worked the best while making any change in the appearance worsened the performance.The techniques used to augment the data during the experiments include horizontal flips, vertical flips, rotations, and affine transformations. Augmentations have been used to achieve rotation invariance as well as to improve generalization while maintaining cytomorphological features in the bone marrow smear images.

- Horizontal Flipping: This technique involves flipping images horizontally, simulating different viewpoints. For example, an image of a cell viewed from the left might look different from the same cell viewed from the right. By flipping images horizontally, we introduce variability in the dataset, enabling the model to learn from different perspectives.
- Vertical Flipping: Like horizontal flipping, vertical flipping involves flipping images vertically to simulate different viewpoints. For instance, an image of a cell viewed from above might look different from the same cell viewed from below. By flipping images vertically, we introduce additional variability in the dataset, allowing the model to learn from diverse viewpoints and improve its ability to generalize to new data.
- Rotation: Adding rotation to the images helps in introducing rotational invariance, which means the model can recognize objects regardless of their orientation. This is particularly useful in scenarios where objects may appear at different angles.

Spatial and positional alterations mimic real life scenarios and enable the model to learn invariant features, therefore improving its robustness to any variations in the input data. In our specific case, we employed horizontal and vertical flipping to simulate the different viewpoints. We also added some rotation to introduce rotational invariance.

### 4.3 Prototypical networks

Meta-learning, a method that focuses on learning from many tasks, encompasses various algorithms like MAML, Reptile, Siamese, Relation, and Prototypical Networks. We have explored various techniques and then compared the initial accuracy to determine the one that was suited the best for our particular use-case.

**Figure 5:**
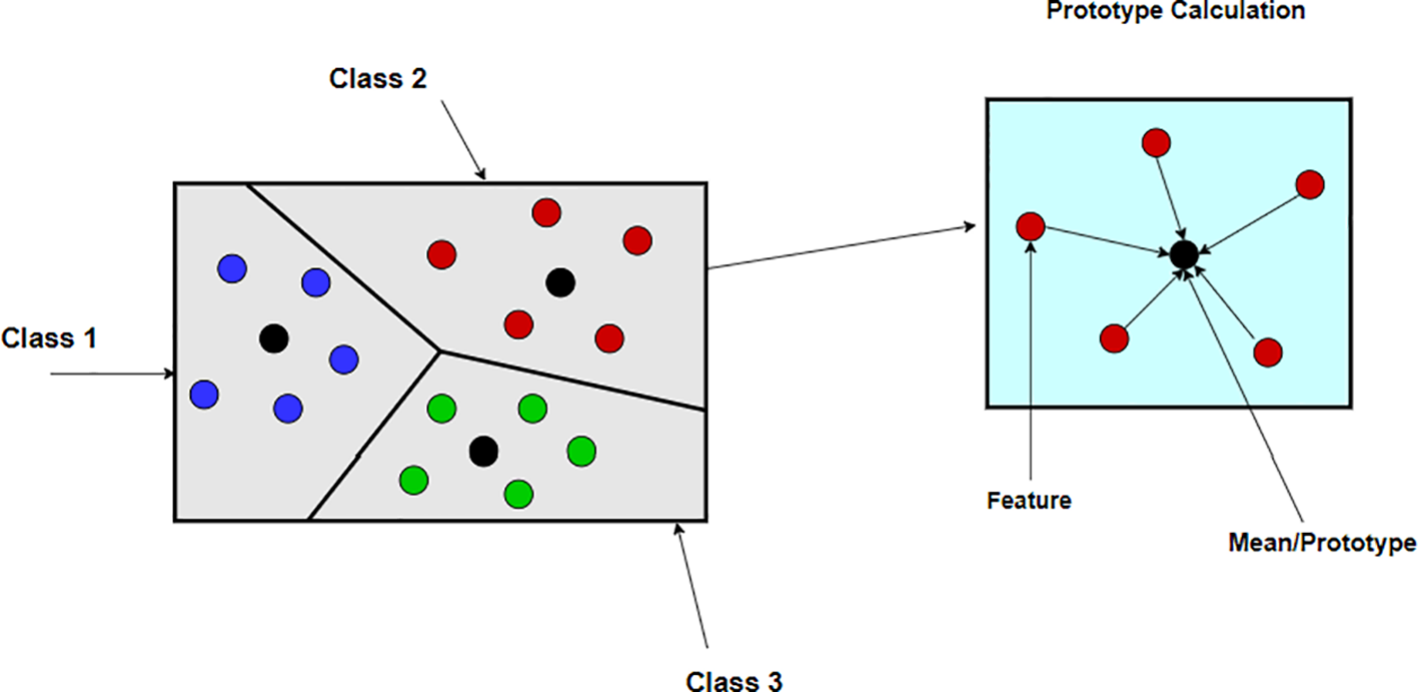
Prototype representation and calculation based on class embeddings.

Meta-learning organizes training around tasks, which consist of a support set (to train the model) and a query set (to predict from). Prototypical Networks, one type of meta-learning method, are used to find prototypes of the support set and query images. The ultimate goal of meta-learning is to find a model that can quickly initialize learning on a new classes. The way Prototypical Networks function is by taking the viewpoint that points cluster around one prototype representation for each class in an embedding space of the images. They do this through a non-linear mapping of inputs to an embedding space using a neural network. One way to visualize this is in **Figure 5**. Specifically, each class’s prototype is computed as the mean vector of the embedded support points belonging to that class.

Equation 1 defines the objective function of the meta-learning framework. Mathematically, the calculation of prototypes can be expressed as:

(1) $$\left(c_{k}\right)=\frac{1}{\left|S_{k}\right|}\sum_{\left(x_{i},y_{i}\right)\in S_{k}}f_{\text{Φ}}\left(x_{i}\right)$$

Where,

*C*_k_ represents the prototype for class k.
*S*_k_ denotes the set of examples labelled with class k.
*f_ϕ_*(*x*_i_) signifies the embedding function parameterized by *ϕ*.

Classification using Prototypical Networks entails determining the nearest class prototype to an embedded query point. This is achieved through a distribution over classes based on SoftMax probabilities. Equation 2 describes the task-specific parameter update process.

(2) $$p_{\text{Φ}}(y=k|x)=\frac{\exp\left(-d\left(f_{\Phi }\left(x\right),C_{k}\right)\right)}{\sum_{k^{\prime }}\exp\left(-d\left(f_{\Phi }\left(x\right),C_{k^{\prime }}\right)\right)}$$

where:

-
$p_{\text{Φ}}(y=k|x)$ represents the probability that the query sample belongs to class *k*.
- *f*_Φ_(*x*) denotes the embedding representation of the query sample.
- *C_k_*represents the prototype of class *k*.
- *d*(·) denotes the distance metric used between embeddings and prototypes.
- *k*^′^ represents all possible classes within the task episode.

Training Prototypical Networks involves minimizing the negative log-probability of the true class via Stochastic Gradient Descent (SGD). Training episodes are constructed by randomly selecting subsets of classes and examples within each class to form support and query sets, respectively. Additionally, analysis reveals that despite the equivalence to a linear model under certain conditions, Prototypical Networks effectively capture non-linearities within the embedding function. Experimental results suggest the advantage of training with a higher number of classes per episode compared to the expected scenario during testing.

The steps involved in this episodic style of training are as follows (**Algorithm 1**):

Algorithm 1 **Training episode generation.**

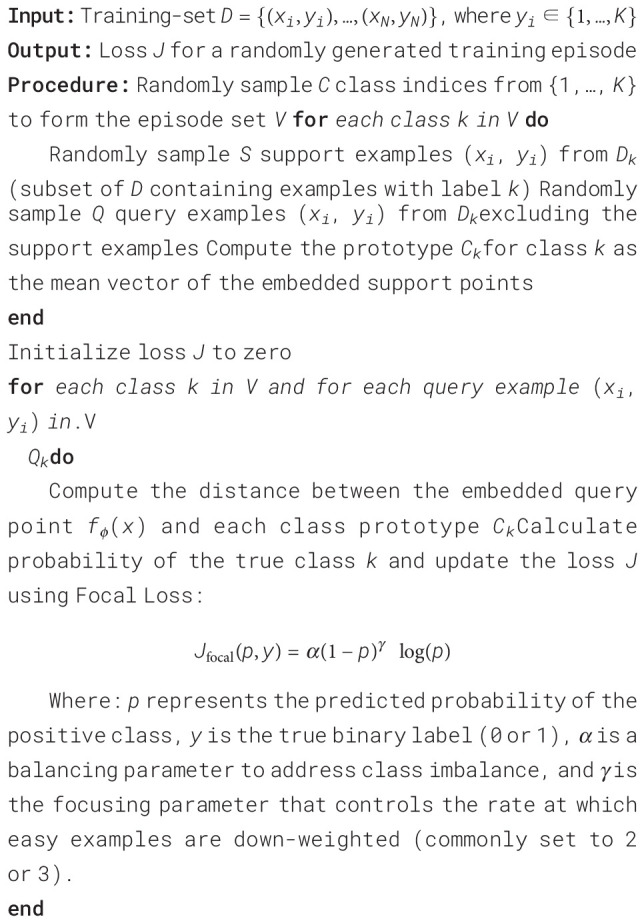

### 4.4 FedAvg algorithm

Federated Learning (FL) provides a way to maintain privacy while eliminating unnecessary communications between clients. FL uses an alternative machine learning approach when drawing from decentralized data sources (nelphones, autonomous vehicles, hospitals etc). it is a smart way to continue to improve an ML model while preserving user privacy. Among FL algorithms, Federated Averaging (FedAvg) is one of the most recognizable algorithms, and a preferred algorithm for many, primarily due to it design and performance.

FedAvg works by alternating stochastic gradient updates at local client nodes and model averaging updates at the server node. This approach to machine learning can take advantage of variation among client data distributions to learn a joint data representation shared on different tasks. There are examples that show better generalization potential for models learned with FedAvg, when evaluating them on newer tasks with several iterations fine tuning. In the weighted averaging form of FedAvg, the algorithm also allows for a way to account for each client contribution to arrive at a fair final model.

In a federated setting with a central server and M clients, each client *i* has a training dataset *D*_i_ comprising *n*_i_ labelled samples drawn from a distribution *D*_i_ over input space X and label space Y. The learning model h*θ*: X→Y is parameterized by *θϵ*RD. The loss of the model on a sample (x, y) is denoted by (h*θ*(x), y). Equation 3 represents the meta-optimization step. The server aims to minimize the average of client losses weighted by the number of samples across clients:

(3) $$\underset{\theta }{\min}\frac{1}{N}\sum_{i=1}^{M}n_{i}f_{i}(\theta )$$

where N and *f*_i_(*θ*) represents the average loss of the model on client *i*’s data. FedAvg executes in rounds, where on each round t, the server uniformly samples a set It of m≤M clients. Each selected client receives the current global parameters *θ*t, performs multiple SGD steps on its local data, and sends the updated parameters back to the server. Equation 4 defines prototype generation for each class. The server then computes the next global iterate as the weighted average of the updates received from clients:

(4) $$\theta_{t+1}=\frac{1}{N_{t}}\sum_{i\in I_{t}}n_{i}\theta_{t},\;i,\tau$$

where *θ*t, i, *τ* represents the parameters after *τ* local updates by client i. The communication efficiency of FedAvg is enhanced by performing *τ*≥2 local updates between communication rounds.

## 5 Experimental setup

**Figure 6:**
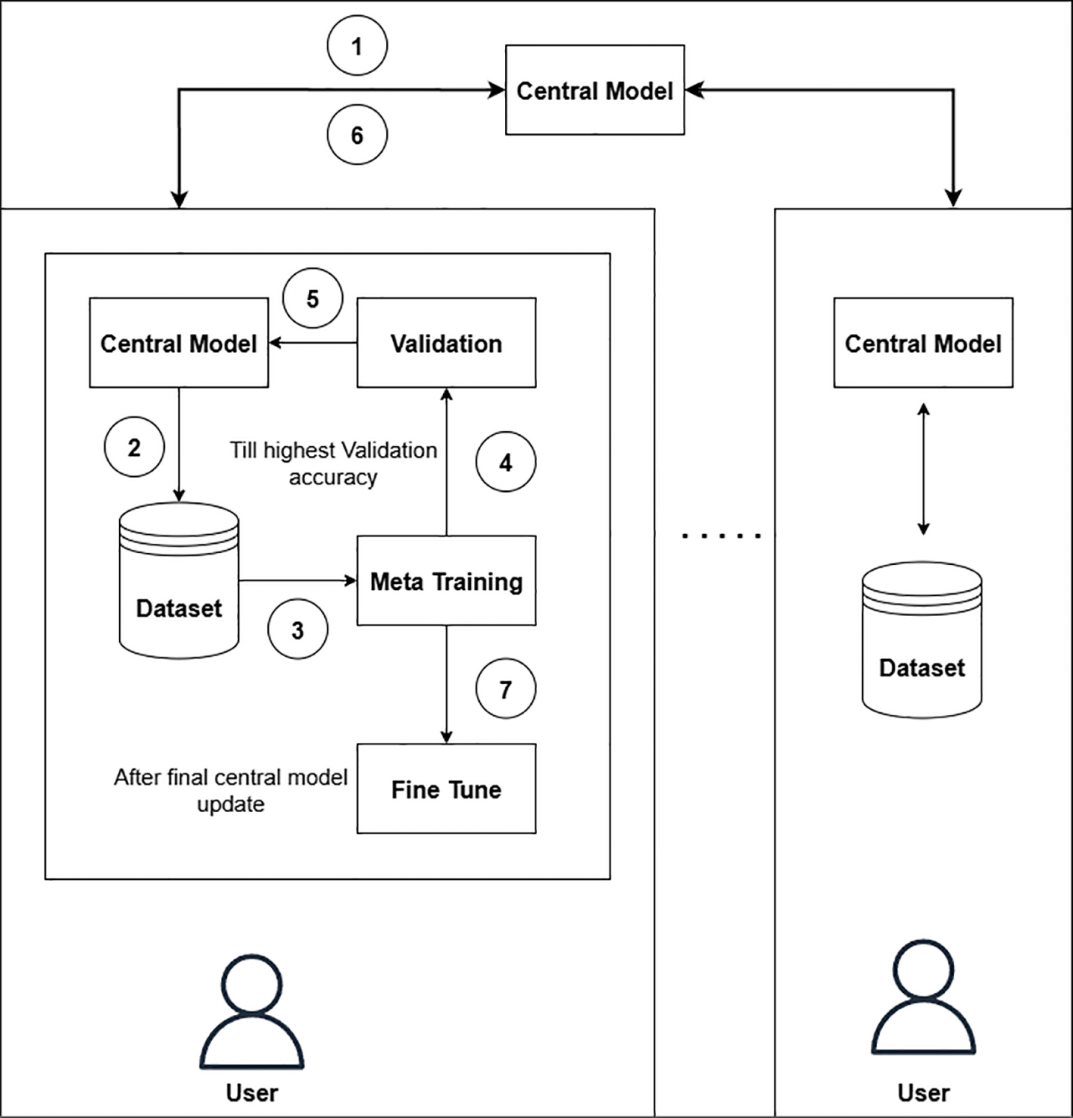
Integrated Model of Smart-FL.

Our approach involves a two-phase strategy, integrating meta-learning with Federated Learning (FL) to enhance model performance in a decentralized environment. This can be explained using the below **Figure 6** where the steps represent:

1. Sharing of the model among clients.
2. Using Local Dataset associated with client.
3. Training the model using meta learning techniques.
4. Iterate for highest validation accuracy.
5. Save the state of the model.
6. Send the model updates back to central sever.
7. Get the averaged model and fine-tune on test set.

### 5.1 Global model training

In our federated learning approach, the training phase involves distributing the global model among the clients, which then individually train the model on their respective datasets. We have integrated this local training of the global model in a meta-training fashion. The global model, a prototypical network with a ResNet18 backbone, serves as the foundation for all clients’ training. For converting our dataset into the taskset with support and query images we have used the Task Sampler from an opensource library called easyFSL. Our objective is to achieve the most adaptable intermediate state for each client. As the training and validation sets contain disjointed classes, there comes a point after several iterations where the model begins to overfit on the training data, hampering its ability to predict on the validation set. Our goal is to pinpoint the optimal state during which the model retains the ability to predict unseen classes with considerable accuracy. These states represent the best adaptable states for each client and will be utilized for weighted averaging. In the propped study the experiment is conducted using 4 clients and distributed varying amounts of data amongst them to simulate a real-life scenario. Since we had GPU constraints on our local device, we had to run our model on Kaggle with T4x2 GPU. We simulated the federated learning by creating a function which defined clients and attached the datasets to them. Then we trained each client and averaged the weights in another function. This allowed us to get the intermediate model which we then used for the second phase of this implementation.

#### 5.1.1 Experimental environment

Based on the limitations of the GPU within the local machine, the experiments were done throughthe Kaggle cloud platform, which supports T4×2 GPUs. The federated learning experiment wascarried out by creating multiple clients, where each client had its own dataset. After training eachclient individually, the averaging of the weights was done using the FedAvg technique to create theglobal intermediate model (**Algorithm 2**).

Algorithm 2 **Smart-FL (Part 1).**

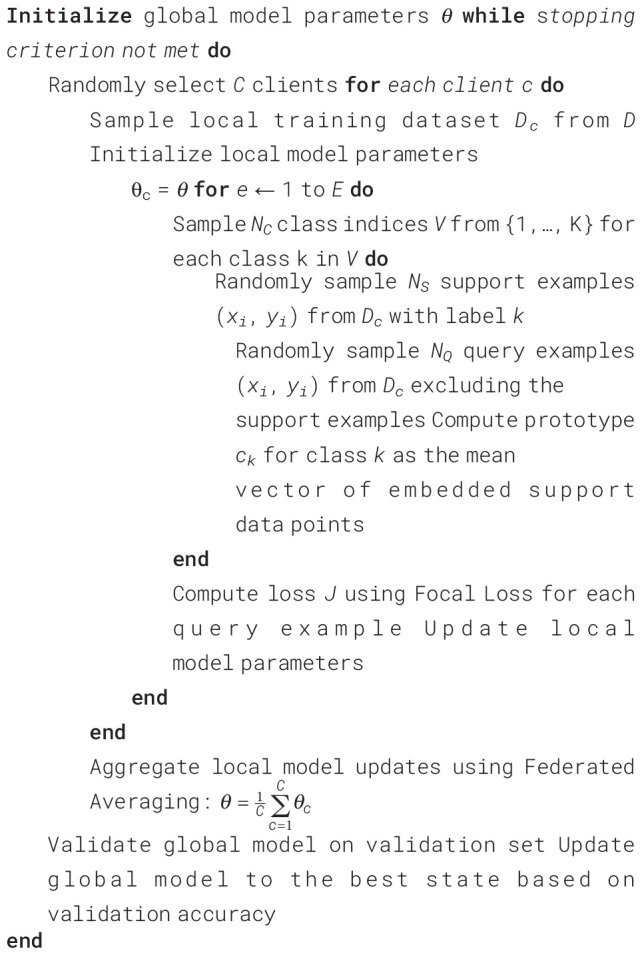

A prototypical network with a pretrained ResNet18 architecture was used in the implementation process. For task generation with support and query images, the Task Sampler was used from the EasyFSL package.

**Figure 7:**
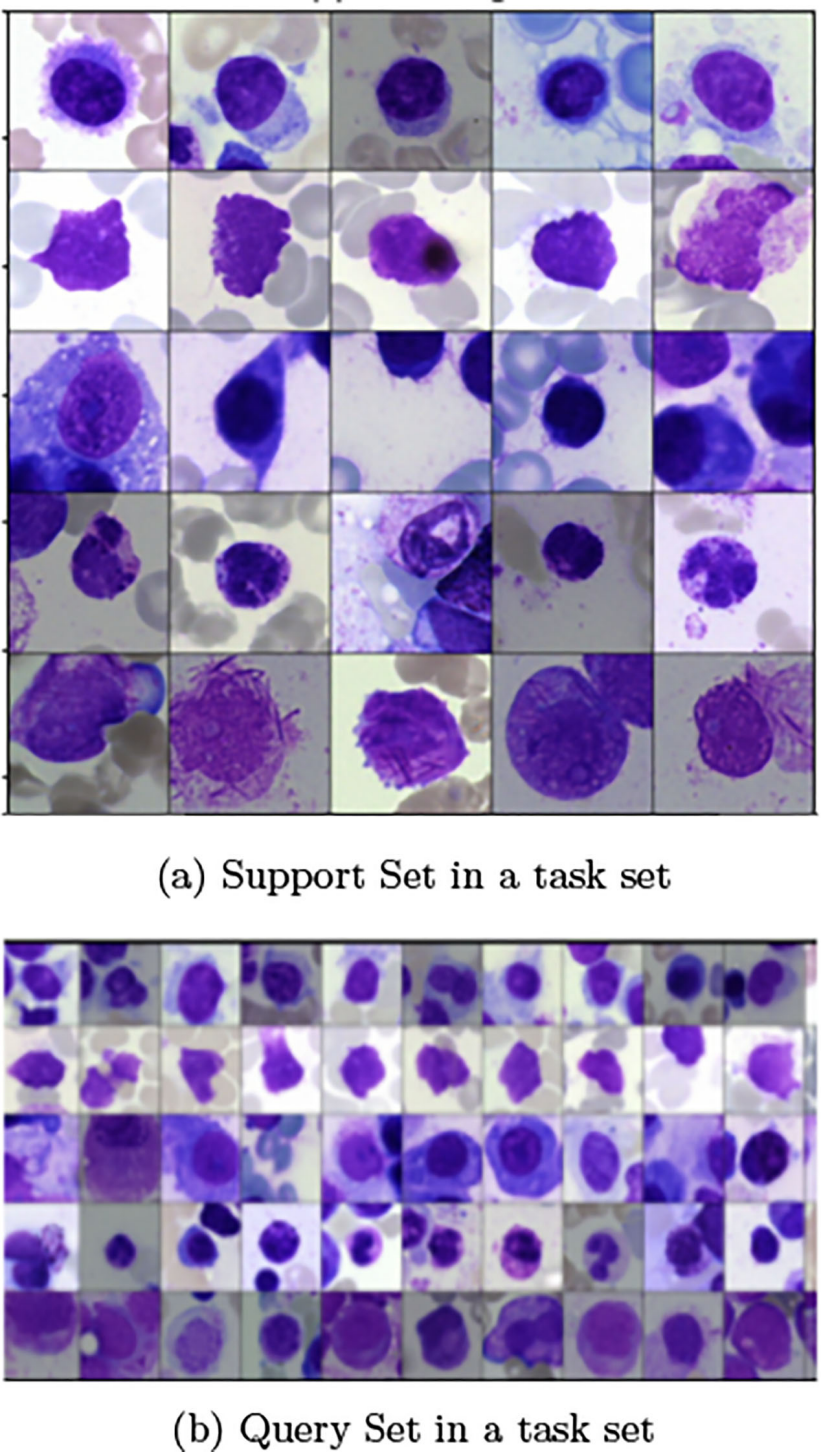
**(a)** Support set and **(b)** Query sets in a task set.

The implementation procedures, such as the client number, the number of classes per episode, support and query images configuration, tasks for training, tasks for validation, optimization procedure, augmentation techniques, and federated learning process, have all been explicitly included in the paper to ensure reproducibility. The evaluation of the proposed Smart-FL framework has also been presented in comparison with previous bone marrow classification methods, which are listed in **Table 1**. Prior research found that there were difficulties in addressing the issue of minority classes and class imbalance, but the current work focuses only on enhancing adaptability and classification accuracy for novel minority classes. **Figure 7** illustrates the structure of a task set, highlighting the support set (**Figure 7a**) and query set (**Figure 7b**).

#### 5.1.2 Implementation details

• Training Classes: 11 training classes and 6 validation classes.

- N-way Classification: Employed with 5 classes.
- Shot and Query: Utilized 5 support images and 10 query images.
- Epochs: Each epoch consists of 500 training tasks and 100 validation tasks.
- Transformations: Basic transformations include horizontal and vertical flips, rotation, and affine transformations for training set.
- Optimization Algorithm: Training conducted using stochastic gradient descent (SGD) with focal loss.
- Learning Rate Scheduler: A multistep learning rate scheduler is employed. **Table 4** summarizes the dataset characteristics and experimental configuration used in this study.

**Table 4:** Dataset and experimental configuration.

Parameter	Description
Dataset Source	“An Expert-Annotated Dataset of Bone Marrow Cytology in Hematologic Malignancies (18)”
Total Patients	945
Dataset Size	Approximately 6.8 GB
Number of Classes	21
Training Classes	11
Validation Classes	6
Testing Classes	3
Image Format	JPG
Backbone Model	ResNet18
Federated Learning Algorithm	FedAvg
Meta-Learning Method	Prototypical Networks
Number of Clients	4
N-Way Classification	5-way
Support Images	5
Query Images	10
Training Tasks per Epoch	500
Validation Tasks per Epoch	100
Fine-Tunning Split	80-20
GPU Platform	Kaggle T4×2 GPU

### 5.2 Local update/fine-tuning

At this stage, we have reached our best adaptable state, which is shared among the clients. In traditional federated learning approaches aimed at achieving personalization, researchers commonly employ local update methods to adapt the model to individual client datasets. **Table 5** presents the distribution of data across the participating clients. However, we have opted for a different approach by utilizing the meta-testing phase or fine-tuning phase. In this phase, we partition the local data into training and testing sets (80–20 split). We then replicate the process conducted during the meta-training phase, but this time, the classes in both sets are the same. For this purpose, we have selected 3 classes, specifically those with the least amount of data, to showcase the efficacy of this approach. **Table 6** provides a comparative analysis of blood marrow classification methods (**Algorithm 3**).

**Table 5:** Client wise data distribution.

Client ID	Images allocated
Client 0	4400
Client 1	3300
Client 2	2200
Client 3	1100

**Table 6:** Comparative study of blood marrow classification.

Classes	Accuracy	Precision	Recall	F1
FGC (Faggot cell), KSC (Smudge cell), LYI (Immature lymphocyte)	96.90%	96.99%	96.90%	96.89%
BLA (Blast), MYB (Myelocyte), PMO (Promyelocyte)	89.33%	89.30%	89.33%	89.29%
ART (Artefact), EBO (Erythroblast), EOS (Eosinophil)	96.57%	96.63%	96.57%	96.57%
LYT (Lymphocyte), MMZ (Metamyelocyte), MON (Monocyte)	91.43%	91.70%	91.43%	91.46%
NGB (Band neutrophil), NGS (Segmented neutrophil), NIF (Not identifiable)	90.17%	90.29%	90.17%	90.18%
BAS (Basophil), HAC (Hairy cell), OTH (Other cell)	92.38%	92.43%	92.38%	92.39%
PEB (Proerythroblast), PLM (Plasma cell), PMO (Promyelocyte)	92.38%	92.41%	92.38%	92.38%
ABE (Abnormal eosinophil), EBO (Erythroblast), KSC (Smudge cell) (2-shot)	96.33%	96.41%	96.33%	96.33%

Algorithm 3 **Smart-FL (Part 2).**

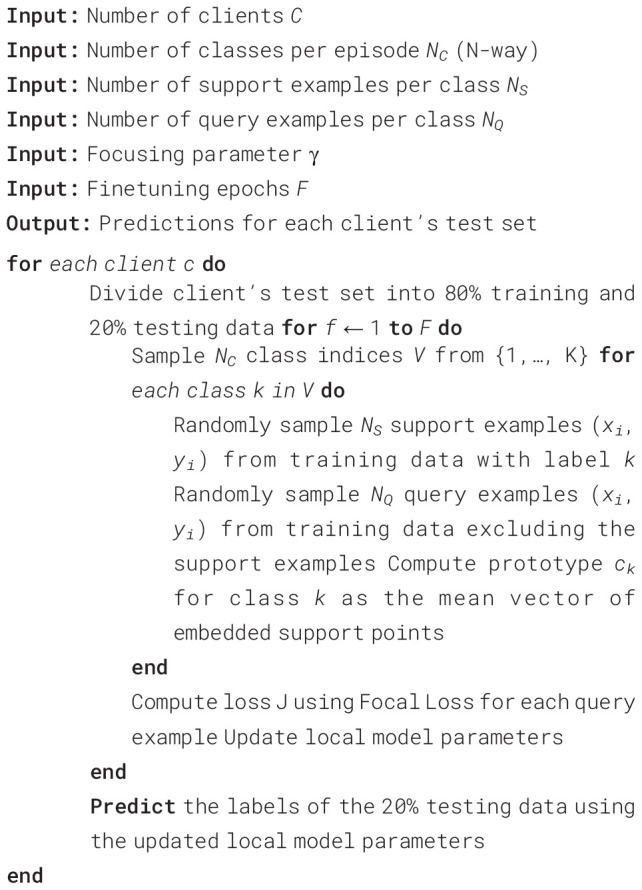

Implementation Details:

- Data Split: Local data partitioned into training and testing sets (80–20 split).
- Class Selection: Utilized 3 classes.
- N-way Classification: Employed with 3 classes.
- Shot and Query: Utilized 3 support images and 7 query images.
- Epochs: Each epoch consists of 10 training tasks, with a validation set of 100 tasks.
- Optimization Algorithm: Consistent usage of the optimization algorithm employed in previous phases.
- Learning Rate Scheduler: Utilized the same learning rate scheduler as in previous phases.

## 6 Results and discussions

**Figure 8:**
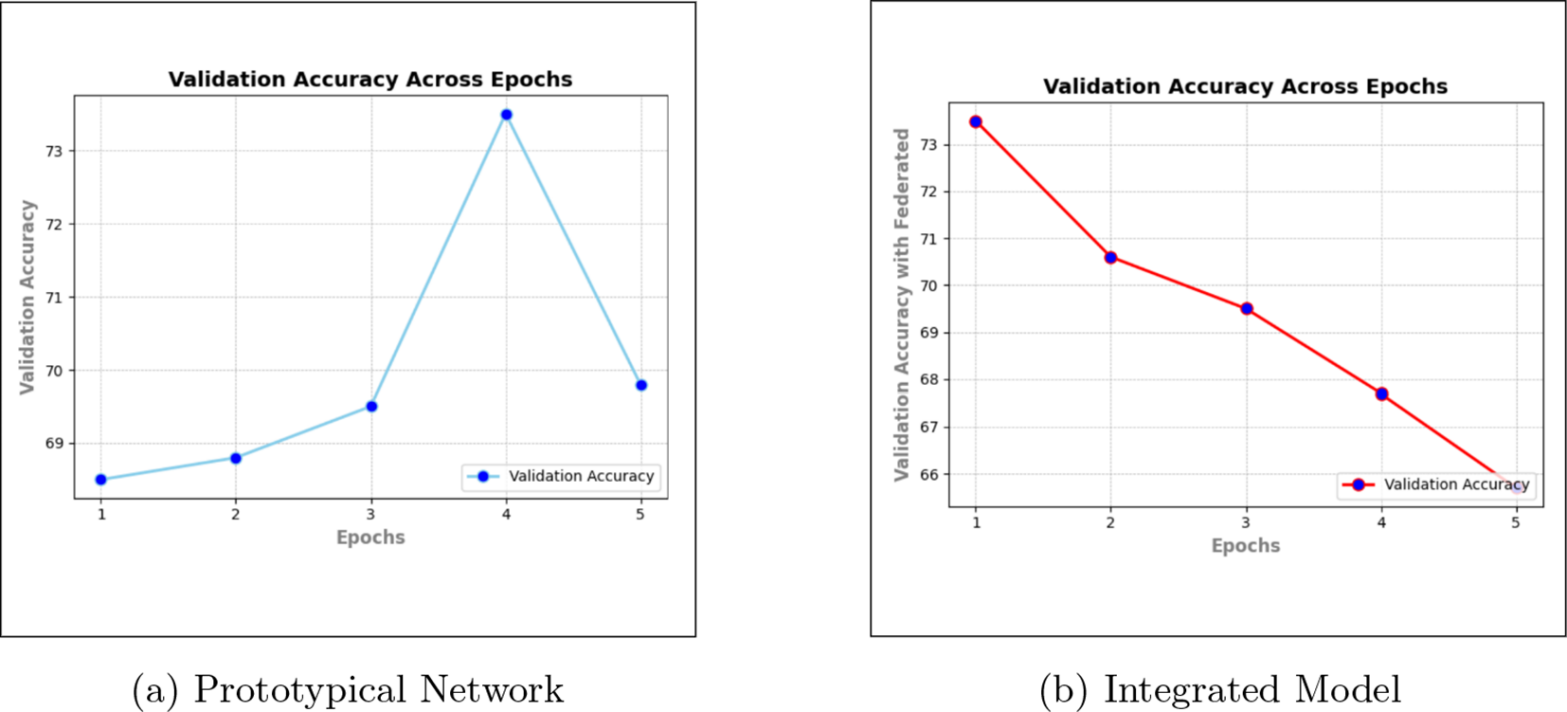
Validation accuracy across epochs. **(A)** Prototypical Network validation accuracy across epochs. **(B)** Integrated Model validation accuracy across epochs.

As outlined in the previous section, during the training of the global model within a federated learning framework, we integrated principles of meta-learning. This approach entailed disseminating the global model among the clients, enabling each client to train autonomously on its respective dataset. The objective of this training procedure was to attain an optimal intermediate state adaptable for all clients. Prior to incorporating federated learning, we achieved this state by the fourth epoch, beyond which the accuracy began to decline. Upon implementing federated learning, we observed that the optimal intermediate accuracy was obtained after a single round, with each client training individually for one epoch as seen in **Figure 8**. We attribute this enhancement to the collaborative nature of model averaging and the ability to leverage larger volumes of data collectively.

In the context of meta-testing, which necessitates the utilization of an n-way classification approach where the number of classes in a task is predetermined, we partitioned the classes designated for fine-tuning into batches of three to yield more precise accuracy metrics. Our objective was to enhance the accuracy for classes with fewer samples available. These classes constituted one group of our experimentation. Additionally, we selected seven sets of three classes each. During our investigation, we noted that certain classes, including those examined by Rohollah Moosavi et al. (14). and other researchers, posed challenges for differentiation as they belonged to the same subcategory. Hence, we ensured the inclusion of this particular set for comprehensive analysis.

### 6.1 Comparative performance discussion

In the experiment, it was noted that the Smart-FL approach was capable of performing better on classes that had fewer samples through the use of federated learning and meta-learning principles.

Firstly, the use of the prototypical network made it possible for the model to adjust well when presented with classes that were not previously seen by the classifier. Secondly, federated averaging increased the adaptability of the global model by using distributed data sets when training.

Along with accuracy, the Smart-FL algorithm was measured on the basis of precision, recall, and F1 score in various bone marrow cell classes. The above-mentioned measures allow obtaining a more accurate evaluation of the classifier’s quality, especially when dealing with imbalance.

Metrics provide a thorough assessment of an image classification model’s performance in properly detecting and classifying an image. Accuracy can be defined as the percentage of images properly classified out of the total number of images. It is measured by dividing the number of correctly classified images by the total number of images. Recall, also called sensitivity, indicates the proportion of relevant instances that were correctly identified by the model in a given class. It can be calculated by the number of true positive predictions divided by the number of true positives and the number of false negatives. Precision reflects the proportion of relevant instances that were correctly identified by the model in a given class. Precision can be measured by the number of true positive predictions divided by the number of true positives and the number of false positives. The F1-score, a harmonic mean of precision and recall, provides a balanced assessment of a model’s performance, especially in cases of class imbalance. Equation 5 computes the distance metric between query samples and prototypes.

Accuracy is computed as:

(5) $$\text{Accuracy}=\;\frac{TP+TN}{TP+TN+FP+FN}$$

where *TP* represents True Positives, *TN* represents True Negatives, *FP* represents False Positives, and *FN* represents False Negatives. Precision is computed as. Equation 6 represents the classification probability calculation.:

(6) $$Precision=\;\frac{TP}{TP+FP}$$

Equation 7 defines the local model update procedure. Recall (Sensitivity) is computed as:

(7) $$Recall=\;\frac{TP}{TP+FN}$$

Equation 8 describes the FedAvg aggregation mechanism. Specificity is computed as:

(8) $$Specificity=\;\frac{TN}{TN+FP}$$

Equation 9 represents the global model update after federated aggregation. F1-score is computed as the harmonic mean of precision and recall, given by:

(9) $$F1-Score=2\;\text{x}\;\frac{Precision\;\times \;Recall}{Precision+\;Recall}$$

As illustrated in the table above, the first row (FGC, KSC, LYI) represents classes with notably sparse examples per class. These particular classes posed significant challenges for previous researchers, with accuracies for those who included these classes reported as low as 20% in some papers. Our approach demonstrates a remarkable improvement in accurately predicting these challenging classes. The second row (BLA, MYB, PMO), as referenced by Rohollah Moosavi et al. (14) and Christian Matek et al. (12)., consists of classes belonging to the same subcategory, which he supplemented by using a tolerant class. This dataset is self-annotated by medical professionals, who themselves encounter difficulty in accurately classifying these three classes. These leaves some room for leeway while classifying these classes. While the inclusion of the tolerant class could potentially elevate our accuracy beyond 92%, we have opted to present the raw values in the table above for transparency. Notably, compared to other researchers whose accuracies range between 30-70%, our approach yields a significant improvement in results. The last row (ABE, EBO, KSC) includes a class ABE which has only 8 samples in the actual dataset so we had to use 2 shot learning for this. Other researchers have gotten accuracies of 2% or have ignored it altogether.

For the remaining classes included, we have observed a satisfactory accuracy, which could potentially be further improved by increasing the number of examples in the n-shot and n-query sets. Increasing the size of these sets could enhance the model’s ability to generalize and classify more accurately.

## 7 Conclusion

In summary, accurate classification of bone marrow cells is crucial for diagnosing hematological disorders, but integrating machine learning into clinical practice faces challenges like data privacy and scarcity. Our study introduces a novel approach using federated learning and meta-learning to tackle these issues. Our goals were to combat data scarcity, address privacy concerns through federated learning, personalize the process for clients, and improve model generalization. We employed ResNet18 and prototypical networks, achieving promising results across four clients. By integrating meta-learning principles into federated learning, we found optimal accuracy after a single round, demonstrating the effectiveness of collaborative model averaging. In the meta-testing approach, we partitioned classes and achieved notable accuracies across various groupings. As shown in previous sections the mentioned approach is able to work well with the presence of similar classes which are closely related to one another. This method also is able to get amazing results using 2-shot on limited samples. These results highlight the potential of this approach in accurately classifying bone marrow cells, even with limited data, promising improved diagnostic capabilities in hematological disorders.

## Data Availability

The datasets presented in this study can be found in online repositories. The names of the repository/repositories and accession number(s) can be found in the article/supplementary material.
